# Inhalable bacteriophage powders: Glass transition temperature and bioactivity stabilization

**DOI:** 10.1002/btm2.10159

**Published:** 2020-04-14

**Authors:** Rachel Yoon Kyung Chang, Philip Chi Lip Kwok, Dipesh Khanal, Sandra Morales, Elizabeth Kutter, Jian Li, Hak‐Kim Chan

**Affiliations:** ^1^ Advanced Drug Delivery Group, School of Pharmacy The University of Sydney Sydney New South Wales Australia; ^2^ Phage Consulting Sydney New South Wales Australia; ^3^ The Evergreen State College Olympia Washington USA; ^4^ Monash Biomedicine Discovery Institute, Infection and Immunity Program and Department of Microbiology Monash University Clayton Victoria Australia

**Keywords:** bacteriophage (phage) therapy, glass transition temperature (*T*_g_), powder, stability, inhalation aerosol

## Abstract

Recent heightened interest in inhaled bacteriophage (phage) therapy for combating antibacterial resistance in pulmonary infections has led to the development of phage powder formulations. Although phages have been successfully bioengineered into inhalable powders with preserved bioactivity, the stabilization mechanism is yet unknown. This paper reports the first study investigating the stabilization mechanism for phages in these powders. Proteins and other biologics are known to be preserved in dry state within a glassy sugar matrix at storage temperatures (*T*_s_) at least ~50°C below the glass transition temperature (*T*_g_). This is because at (*T*_g_ − *T*_s_) >50°C, molecules are sufficiently immobilized with reduced reactivity. We hypothesized that this glass stabilization mechanism may also be applicable to phages comprising mostly of proteins. In this study, spray dried powders of Pseudomonas phage PEV20 containing lactose and leucine as excipients were stored at 5, 25 or 50°C and 15 or 33% relative humidity (RH), followed by assessment of bioactivity (PEV20 stability) and physical properties. PEV20 was stable with negligible titer loss after storage at 5°C/15% RH for 250 days, while storage at 33% RH caused increased titer losses of 1 log_10_ and 3 log_10_ at 5 and 25°C, respectively. The plasticizing effect of water at 33% RH lowered the *T*_g_ by 30°C, thus narrowing the gap between *T*_s_ and *T*_g_ to 19–28°C, which was insufficient for glass stabilization. In contrast, the (*T*_g_ − *T*_s_) values were higher (range, 46–65°C) under the drier condition of 15% RH, resulting in the improved stability which corroborated with the vitrification hypothesis. Furthermore, phage remained stable (≤1 log_10_) when the (*T*_g_ − *T*_s_) value lay between 26–48°C, but became inactivated as the value fell below 20°C. In conclusion, this study demonstrated that phage can be sufficiently stabilized in spray dried powders by keeping the (*T*_g_ − *T*_s_) value above 46°C, thus supporting the vitrification hypothesis that phages are stabilized by immobilization inside a rigid glassy sugar matrix. These findings provide a guide to better manufacture and storage practices of inhaled phage powder products using for translational medicines.

**Abbreviations**
DSC: differential scanning calorimetry
RH: relative humidity
TGA: thermal gravimetric analysis.
XRD: X‐ray diffraction pattern

## 1 INTRODUCTION

Recent heightened interest in inhaled bacteriophage (phage) therapy for bacterial pulmonary infections has led to the development of phage powder aerosol formulations. Phages are viruses that specifically infect and kill particular strains of bacteria so they provide a potential solution to multi‐drug resistant bacterial infections. Delivering them as aerosols can achieve direct targeting in the lungs. The feasibility of bioengineering phages into inhalable powders by spray drying has been demonstrated in vitro^1^, ^2^, ^3^, ^4^, ^5^, ^6^ along with efficacy studies in vivo.^7^, ^8^ In the production process, it is essential to preserve phage bioactivity in the dry state by suitable excipients. Disaccharides such as trehalose, sucrose, and lactose are reported to provide protection to phages during drying and subsequent storage.^1^, ^2^, ^3^, ^4^, ^5^, ^6^ This concept of sugar‐imparted phage protection was originated from protein stabilization mechanisms.^9^, ^10^ There are two hypotheses on the mechanism of stabilizing proteins by sugars in the solid state, namely, water replacement and vitrification.^9^, ^10^ Since proteins are a major constituent of phages, these stabilization hypotheses may also be applicable to phages.^3^, ^4^, ^11^

In the water replacement hypothesis, a protein molecule maintains its conformation by interacting with water molecules through hydrogen bonding. While this interaction is lost during the drying process, the hydroxyl groups of sugar molecules replace the hydrogen bonds of water and maintain the protein native structure.^12^ Hydrogen bonding between protein and sugar is more likely to occur when the sugar is in the amorphous rather than crystalline state. The sugar molecules interact with each other in the crystalline state instead of interacting with the protein molecules. Mannitol is a good example of a polyol that tends to form crystalline structures after spray drying from solution. Crystallization reduces the molecular interactions (hydrogen bonding) between the sugar and protein, thereby denaturing the protein during dehydration.^13^, ^14^ Crystalline mannitol thus compromised phage stability in an earlier study,^1^ whereas amorphous sugars preserved phage activity when co‐spray dried.^1^, ^2^, ^5^

The vitrification hypothesis states that proteins can be immobilized inside a rigid, amorphous glassy sugar matrix. This dramatically slows down translational molecular movements and subsequent degradation of proteins. An amorphous sugar glass is characterized by a glass transition temperature (*T*_g_). *T*_g_ is the temperature at which the amorphous sugar transitions between a glassy state and a rubbery state. At temperatures above *T*_g_, amorphous glassy sugars become rubbery and then crystallization most likely occurs. This recrystallization is detrimental to the stability of proteins.^15^, ^16^ Protein degradation rate is much slower in the glassy state due to reduced molecular mobility. It was proposed that negligible molecular mobility is achieved by storing at 50°C below the *T*_g_.^17^ In addition, the presence of water can decrease the *T*_g_ of sugar glasses through its plasticizing effect. The higher hygroscopicity of amorphous sugars can increase the likelihood of this.^18^

The two hypotheses have a common ground of protein structure preservation by minimizing the molecular mobility of proteins to prevent conformational changes.^15^ Grasmeijer et al investigated where the two hypotheses stand in protein stabilization using a model protein, alkaline phosphatase, spray dried with trehalose or inulin.^10^ Ammediol was incorporated in the protein‐sugar matrix to act as a plasticizer and adjust the *T*_g_. Protein stability was dramatically improved by increasing the *T*_g_ so that it became 20–30°C higher than the storage temperature (*T*_s_). The stability could not be further improved beyond this temperature, instead, it was more dependent on the sugar used with trehalose providing superior protein stability. Being a small molecule, trehalose can closely fit the irregular protein surface and form more hydrogen bonds with the protein. The authors concluded that vitrification plays a vital role in protein stabilization when *T*_g_ is 10–20°C above the *T*_s_. However, proteins become almost immobile at a higher *T*_g_ and water replacement mechanism becomes the overriding protein stabilization mechanism. Many other studies have attempted to understand protein stabilization mechanism in the solid state.^10^, ^12^, ^19^, ^20^, ^21^ However, only a limited number of studies have explored phage stabilization in spray dried powders.^3^, ^4^, ^22^

A study by Vandenheuvel et al showed recrystallization of spray dried phage formulation containing trehalose after storage at 54% RH with up to 3 log_10_ titer reduction.^22^ Recrystallization of amorphous trehalose was confirmed by X‐ray diffraction (XRD) and an absence of *T*_g_ as measured by differential scanning calorimetry (DSC). Spray dried phage powders stored in dry condition did not show changes in *T*_g_ or crystallinity, and the phage titer was retained. Similarly, Leung et al demonstrated that spray dried phage formulations containing trehalose and leucine remained stable in dry condition for 1 year at ambient temperature.^3^, ^4^ While all these studies highlighted the importance of amorphous sugar and storage humidity on phage stability in powder formulations, the interplay of *T*_g_ and *T*_s_ on phage stability is unknown. In this study, we explored the vitrification hypothesis with the aim to understand if the same stabilization mechanism may also be applicable to phages which contain proteins as the major constituent. To this end, we assessed the role of *T*_g_ and storage temperature and humidity on phage bioactivity.

## 2 EXPERIMENTAL SECTION

### 2.1 Materials

An anti‐pseudomonal lytic phage PEV20 was isolated and characterized by the Kutter Lab (Evergreen State College, WA). Phage propagation and purification work for this study was kindly performed by AmpliPhi Australia. Phages (10^10^ pfu/ml) were stored in a phosphate buffered saline (0.01 M phosphate buffer, 0.0027 M KCl and 0.137 M NaCl), with pH adjusted to 7.4. *Pseudomonas aeruginosa* dog‐ear strain PAV237 was used as a reference bacterial strain to assess phage titer. Lactose monohydrate (DFE Pharma, Goch, Germany) and L‐leucine (Sigma‐Aldrich, NSW, Australia) were used as excipients for spray drying PEV20. These two excipients can protect and stabilize phage particles from the stresses of spray drying with minimal titer loss, and form partially‐crystalline and partially‐amorphous particles.^1^, ^7^ Furthermore, our recent long‐term stability study has shown that lactose, despite being a reducing sugar, provides superior phage protection to trehalose.^23^ Two formulations with different excipient ratios were studied: 80% (wt/wt) lactose and 20% (wt/wt) leucine, and 50% (wt/wt) lactose and 50% (wt/wt) leucine.

### 2.2 Atomic force microscopy

PEV20 phage suspension was diluted 1:10 with ultrapure water. Diluted phage suspension was drop casted (5 μl) on a silicon wafer and the phages were allowed to adhere on the surface for 3 min. Excess liquid was removed from the silicon wafer and the sample was left to air‐dry for 30 min prior to image acquisition. Images were acquired using nanoIR atomic force microscopy (Anasys Instruments) operated in tapping mode using a silicon nitride cantilever with a spring constant of 40 N/m (EXT125, AppNano, Mountain View, CA) at a scan rate of 0.50 Hz and a resolution of 500 × 200 points. The scanned images were processed using Analysis Studio (Anasys Instruments).

### 2.3 Transmission electron microscopy

The morphology of phage PEV20 examined by transmission electron microscopy. Samples were prepared on carbon copper grids 200 mesh. Poly‐l‐lysine solution (0.1%) was drop casted on a carbon copper grid (200 mesh) for 1 min and then blotted with a filter paper. Then, the phage lysate was drop casted on top of the grid for 30 min with any excess sample blotted. The samples were negatively stained with 1% phosphotungstic acid (pH 7.4) for 20 s. Excess stain was blotted and the grids were left to air‐dry overnight. A JEOL‐JEM 1400 microscope (JEOL, Japan) was used to collect images at ×40 k magnification.

### 2.4 Phage powder production

Spray dried phage powders were prepared as per our previous study.^1^ Briefly, the liquid feed composed of 0.5 ml of phage suspension (10^10^ pfu/ml) and 50 ml of excipient solution (pH 7.4) at a total solid content of 25 mg/ml. The phage titer was determined prior to spray drying using a standard plaque assay (see below). The mixtures were spray dried using a Büchi 290 spray dryer (Buchi Labortechnik AG, Flawil, Switzerland) coupled with a conventional two‐fluid nozzle for atomization. Spray drying conditions were as follows: feed rate of 1.9 ml/min, atomizing airflow of 742 L/hr, aspiration rate of 35 m^3^/hr, inlet temperature of 60°C and outlet temperature of 40–41°C. The produced powders were aliquoted into scintillation vials for storage. The phage titer in the powders was determined by plaque assay (see below) after reconstitution in phosphate‐buffered saline.

### 2.5 Plaque assay

A double‐layer agar plate was prepared by mixing 0.2 ml of an overnight culture of *P. aeruginosa* dog‐ear strain PAV237 (approximately 2 × 10^8^ colony forming units) with 5 ml of 0.4% nutrient broth top agar and overlaying onto a 1.5% nutrient agar plate. Then 10 μl of serially diluted phage suspension was dropped on top of the top agar plate, left to dry for 15 min and incubated at 37°C for 18 hr. The assay was independently conducted three times. Phages were considered stable if the titer loss was less than log_10_ 0.5. Student *t* test was used to examine the statistical significance of the data. The null hypothesis was rejected if the *p* value was <.05.

### 2.6 Storage conditions

Aliquoted phage powders in vials (200 mg) were stored uncapped at 5, 25, or 50°C and 15% RH or 33% RH for 3 days to allow equilibration of powders at the specified conditions. The phage powders were further stored at 5 or 25°C and 15% RH or 33% RH up to 250 days. A humidity‐controlled acrylic box (15 or 33% RH) was used to avoid unintended moisture uptake of the powders during handling of phage powders. Storage condition of 50°C/33% RH was excluded from this study as significant titer loss within a few days. Storage conditions deviated from the ICH guidelines^24^ as this study focused on exploring the mechanistic basis of phage stability in powder formulations. To achieve this aim, we chose conditions that could sufficiently induce differential changes in the *T*_g_ between the formulations. The 15% RH was produced using silica beads and 33% RH a saturated magnesium chloride solution. The solution was prepared by dissolving excess magnesium chloride in warm distilled water at 40°C with stirring until no more salt could dissolve. The solution was left to cool to ambient temperature and then allowed to set in an airtight container for 24 hr before use.

### 2.7 Differential scanning calorimeter

A differential scanning calorimeter (DSC; Mettler Toledo, Greifensee, Switzerland) was used to assess the thermal properties of the phage powders. A small amount of powder (5 ± 1 mg) was weighed into an aluminum crucible and then crimped to a perforated lid. Powders stored at 15% RH were weighed and crimped inside a dry box (12–17% RH) and those stored at 33% RH in a RH‐controlled box (30–36% RH) to minimize exposure to ambient conditions. The crucible was heated from 22 to 150°C at a rate of 10°C/min under 250 cm^3^/min nitrogen purge. DSC curves were analyzed using STARe software (Mettler‐Toledo, Switzerland). *T*_g_ was determined by fitting straight lines to the DSC curve before, during, and after the transition, and then taking an average of the onset and midpoint temperatures. The assay was independently conducted three times.

### 2.8 X‐ray diffraction

XRD pattern of the samples were recorded using an X‐ray diffractometer (X'Pert PRO; PANalytical, Almelo, The Netherlands) under ambient conditions. Samples were packed in glass capillary tubes with an internal diameter of 1.0 mm (WJM‐Glas, Berlin, Germany) and subjected to Cu Kα radiation at 40 mA and 45 kV. The scattered intensity was collected by a detector for a 2*θ* range of 3°–50° at an angular increment rate of 0.028° 2*θ*/s.

## 3 RESULTS AND DISCUSSION

**FIGURE 1:**
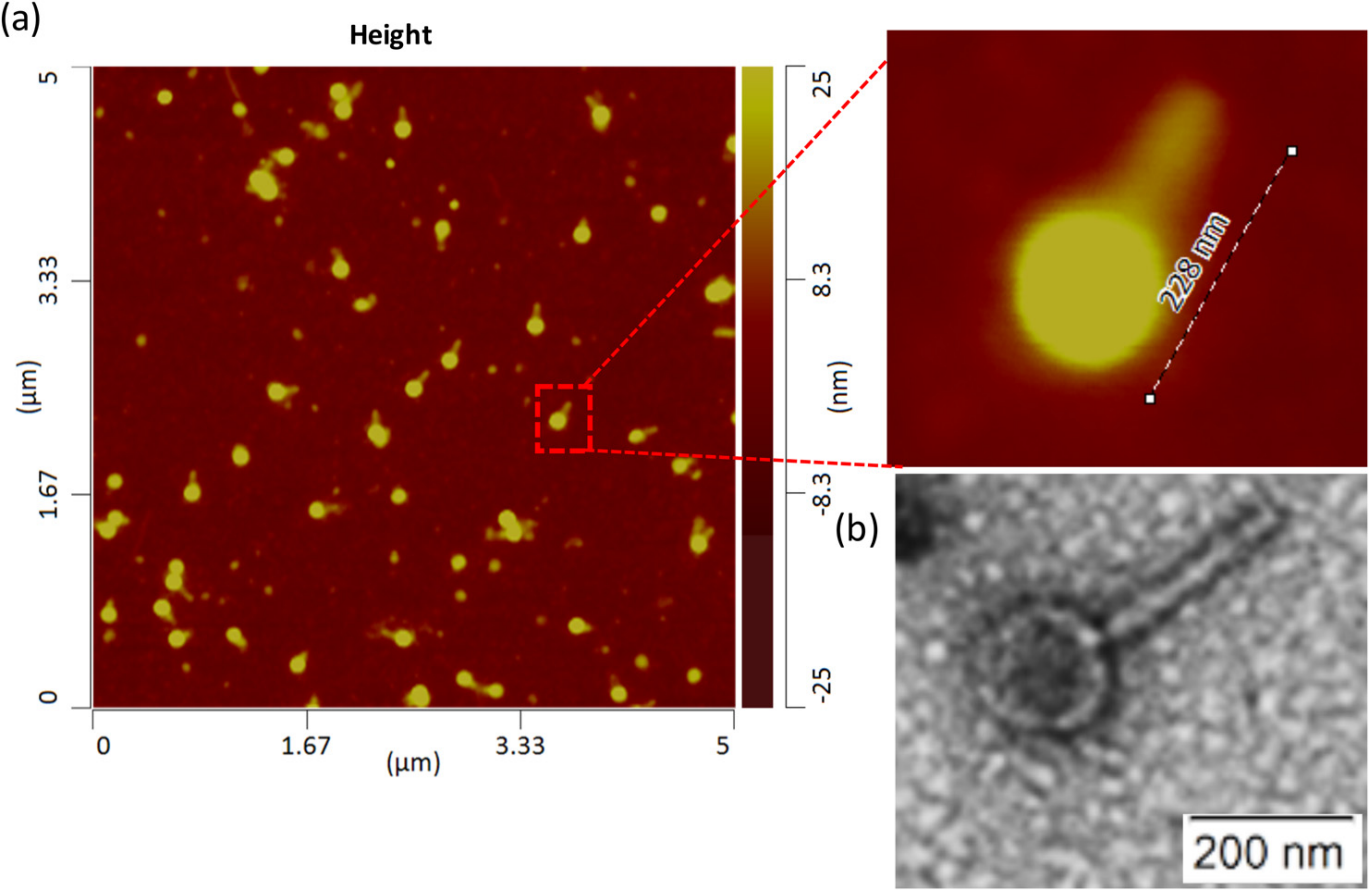
Atomic force microscopic image of PEV20 phages immobilized on a silicon wafer with a zoomed in insert showing individual phages (a) and a transmission electron microscopic image (b)

**FIGURE 2:**
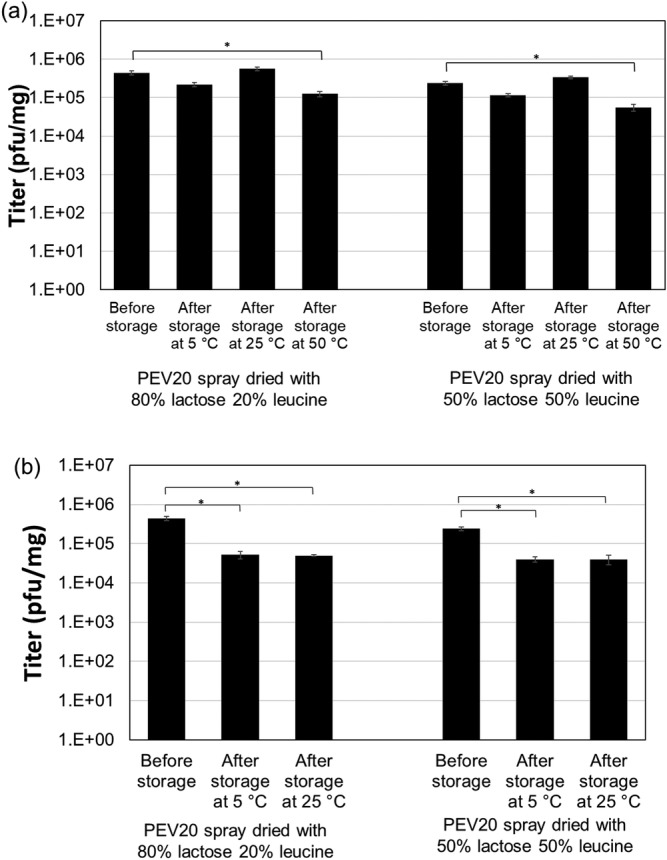
Titer of PEV20 powders containing 80% lactose/20% leucine or 50% lactose/50% leucine after storage at 5, 25, or 50°C and 15% RH (a) or 33% RH (b) (*n* = 3). Data are presented as mean ± SD in the graphs. Asterisk represents significant difference; *p* value <.05

PEV20 phages in the lysate was morphologically intact with the head attached to the tail with a head size of 120 nm and tail length of 130 nm (Figure 1). The biological activity of these phages were largely retained after spray drying with a total of 0.1 log_10_ titer reduction per mg. The final titer of PEV20 was 4.4 × 10^5^ pfu/mg in the spray dried powder containing 80% lactose and 20% leucine (Figure 2a).

The phage powder was stored at 5, 25, or 50 and 15% RH for 3 days to allow equilibration at the specified conditions. Phages remained stable at 5°C (0.2 ± 0.0 log_10_ titer loss) and 25°C (no change), whereas storage at an elevated temperature of 50°C caused 0.7 ± 0.1 log_10_ titer loss (Figure 2a).

**FIGURE 3:**
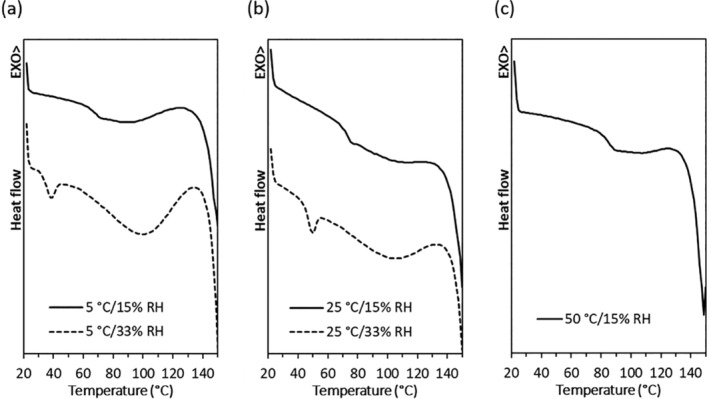
DSC curves of spray dried PEV20 powder containing 80% lactose and 20% leucine after a three‐day storage at 5°C (a), 25°C (b), or 50°C (c) and 15% or 33% RH

**FIGURE 4:**
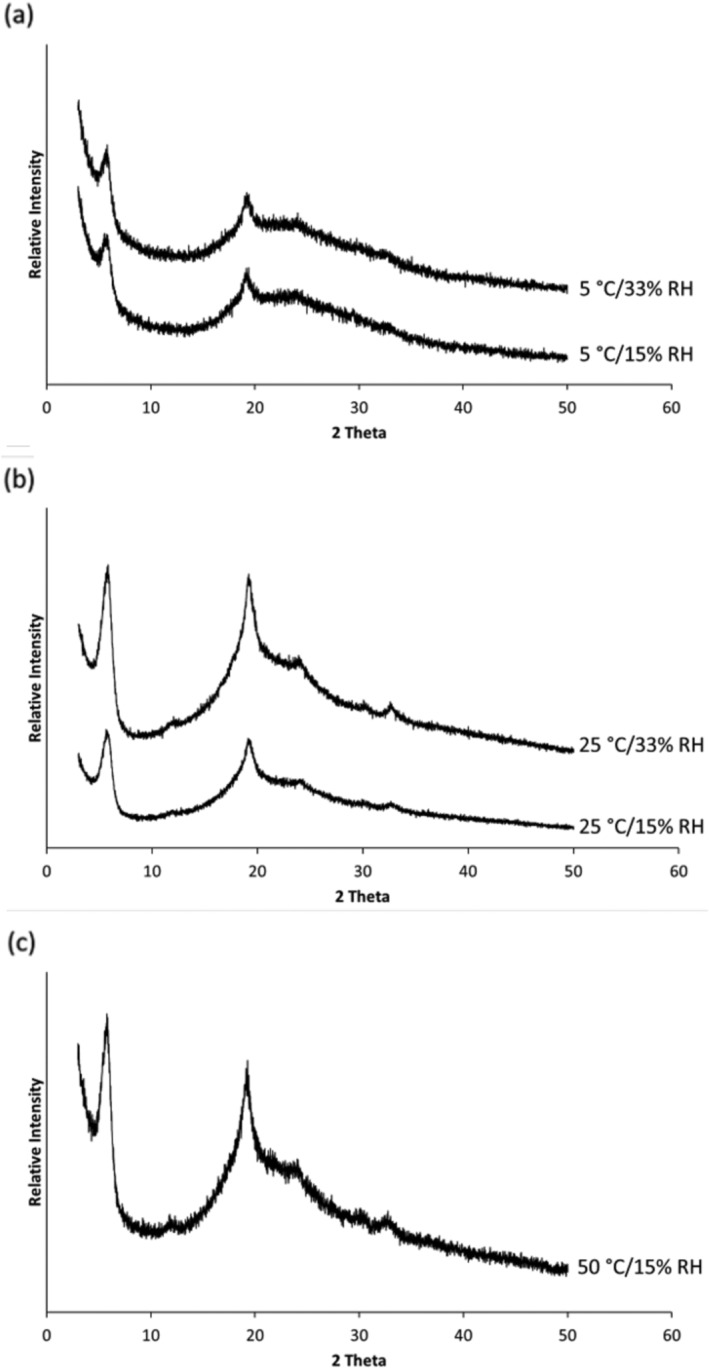
X‐ray powder diffraction patterns of spray dried PEV20 powders containing 80% lactose and 20% leucine after storage at 5°C (a), 25°C (b), or 50°C (c) and 15% or 33% RH

The *T*_g_ of the powders ranged from 66–71°C after a 3‐day storage at 5 or 25°C/15% RH, while storage at 50°C/15% RH shifted the *T*_g_ to 82°C (Figure 3). The increase in the *T*_g_ may be due to thermal annealing at the higher temperature. This phenomenon has been observed in other solid materials, even when the storage temperature was below the *T*_g_.^25^, ^26^, ^27^ XRD data (Figure 4 and Figure S1) showed that all the phage powders stored at <25°C/15% RH remained partially crystalline with diffraction peaks from crystalline leucine at 6°, 19°, and 34°.^1^, ^2^ As the phage content in the spray dried powder is extremely low compared to excipients, the presence of phages does not affect the XRD pattern.^1^ After storage at 50°C, *T*_g_ increased by 10°C but the temperature gap between *T*_s_ and the *T*_g_ was only 32°C (Figure 3c and Table 1), and phages became unstable within 3 days (Figure 2a). In addition, chemical degradation of the phage powders may have occurred at 50°C.

**TABLE 1:** *T*_g_ and subsequent gap between *T*_g_ and *T*_s_ of spray dried phage powders after a 3‐day storage at 5, 25, or 50°C and 15% RH or 33% RH

Excipient composition	Storage RH (%)	Stored at 5°C		Stored at 25°C		Stored at 50°C	
		*T*_g_ (°C)	*T*_s_ − *T*_g_ (°C)	*T*_g_ (°C)	*T*_s_ − *T*_g_ (°C)	*T*_g_ (°C)	*T*_s_ − *T*_g_ (°C)
80% lactose 20% leucine	15	66 ± 0.3	61	70 ± 0.5	45	82	32
50% lactose 50% leucine		69 ± 1.1	64	73 ± 0.1	48	86	36
80% lactose 20% leucine	33	30 ± 0.4	25	44 ± 0.6	19		
50% lactose 50% leucine		32 ± 0.7	27	46 ± 0.1	21		

In comparison, phages remained stable at <25°C/15% RH (Figure 2a) while the temperature gap between *T*_g_ and *T*_s_ (i.e., *T*_g_ − *T*_s_) was greater, >46°C (Table 1). Due to the nature of the plaque assay technique, a titer variation of 0.5 log_10_ is considered an insignificant change.^1^ As a rule of thumb, *T*_s_ should be at least 40–50°C below the *T*_g_ to substantially slow down the translational molecular mobility to enhance protein stability.^17^ Low (*T*_g_ − *T*_s_) value at 50°C/15% RH could explain the phage instability followed by a loss of bioactivity.

There is a possibility that the observed titer loss could be due to protein unfolding and degradation caused by the high temperature of 50°C. At present, it is not possible to study phage protein unfolding using conventional spectroscopic analyses such as Fourier‐transform infrared or Raman spectroscopies. These techniques are not sufficiently sensitive to detect phage content in the powder. To observe the effect of *T*_g_ on phage stability without the influence of high temperature, the phage powder was stored at 33% RH in an effort to lower the *T*_g_. Water is a strong plasticizer that can reduce *T*_g_ and increase local molecular mobility.^28^ At an elevated RH (33%), the powder will absorb water from the environment and consequently lower the *T*_g_. Storage at 5°C/33% RH and 25°C/33% RH caused 0.9 ± 0.0 and 1.1 ± 0.0 log_10_ phage titer reductions, respectively (Figure 2b). While phages remained stable at 5 and 25°C under dry (15% RH) condition (Figure 2a), a higher RH of 33% caused the phages to become unstable. Furthermore, DSC measurements showed that the *T*_g_ was decreased after storage at the higher RH. After storage at 5°C/33% RH, the observed *T*_g_ was 30°C (Figure 3) with a low (*T*_g_ − *T*_s_) value of 25°C (Table 1). Storage at 25°C/33% RH further reduced the (*T*_g_ − *T*_s_) value down to 19°C (Table 1). XRD confirmed that the powders remained partially crystalline due to the presence of crystalline leucine (Figure 4). Phage stability in spray dried powder is compromised when (*T*_g_ − *T*_s_) was <40°C because local molecular mobility was increased,^20^ which can accelerate chemical degradation of proteins, such as deamination, oxidation, and covalent aggregation.^29^, ^30^ Increased local mobility could also impact phage stability by increasing the mobility of phages, causing conformational changes and subsequent loss of biological activities. The results from this study showed that Hancock's proposition^17^ on ideal storage condition for protein powder formulations (*T*_g_ − *T*_s_ > 50°C) also applies to phage powders, which may be attributed to vitrification.

**FIGURE 5:**
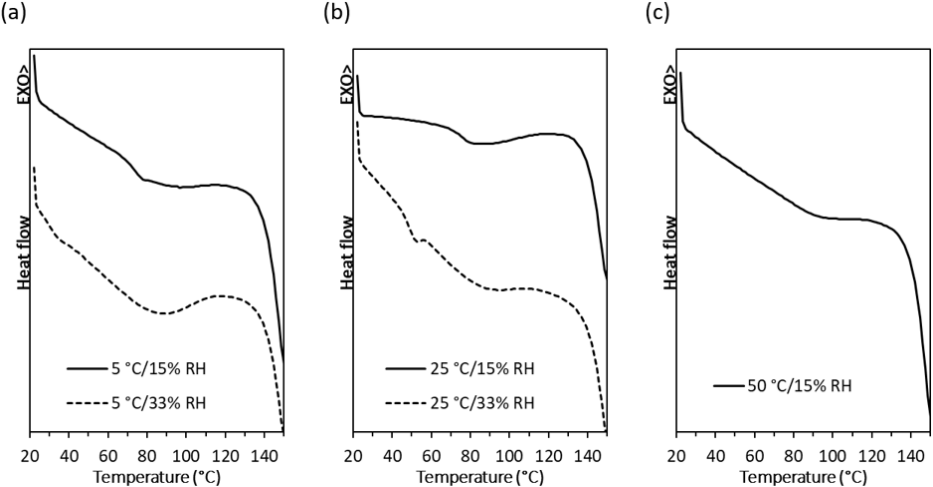
DSC curves of spray dried PEV20 powder containing 50% lactose and 50% leucine after a three‐day storage at 5°C (a), 25°C (b), or 50°C (c) and 15% or 33% RH

**FIGURE 6:**
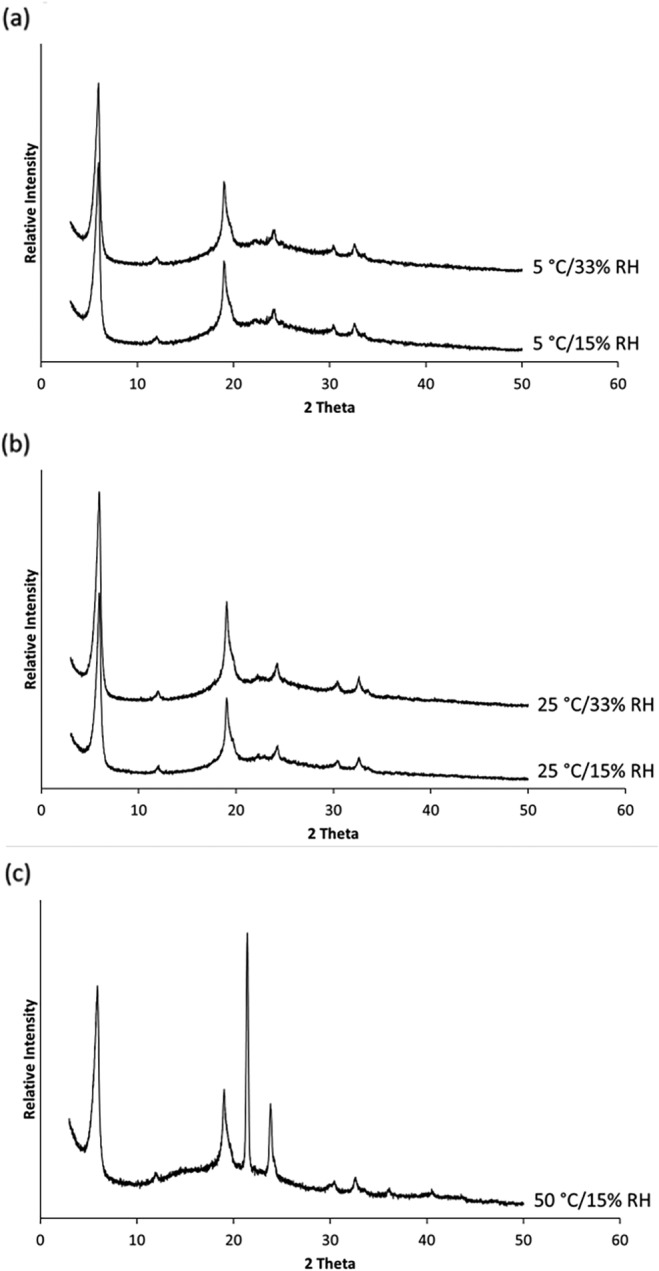
X‐ray powder diffraction patterns of spray dried PEV20 powders containing 50% lactose and 50% leucine after storage at 5°C (a), 25°C (b), and 50°C (c) with 15% or 33% RH

Similar results were obtained for spray dried phage powders containing 50% lactose and 50% leucine. Phage powder was produced with minimal titer loss of 0.4 log_10_ per mg, resulting in a final titer of 2.4 × 10^5^ pfu/mg (Figure 2). No major differences in phage stability were observed as compared with phage powder containing 80% lactose and 20% leucine (Figure 2). This indicated sufficient phage stabilization in the spray dried powders. Furthermore, no notable differences in *T*_g_ (Figure 5) and XRD data (Figure 6 and Figure S1) were observed, except for those stored at a high temperature. After a 3‐day storage at 50°C/15% RH, sharper and stronger diffraction peaks were observed with an additional prominent peak at 21° that belongs to crystalline lactose,^31^ suggesting recrystallization of amorphous lactose (Figure 6c). A prominent diffraction peak at 21° that belongs to anhydrous β‐lactose crystals^31^ appeared after storage at 50°C. However, this was only a partial recrystallization of lactose with some amorphous content remaining in the phage powder, as *T*_g_ was still measurable. Nonetheless, recrystallization is detrimental for phage stability as it causes loss of molecular interactions between phages and lactose as well as shear stress to the phage.^32^

**FIGURE 7:**
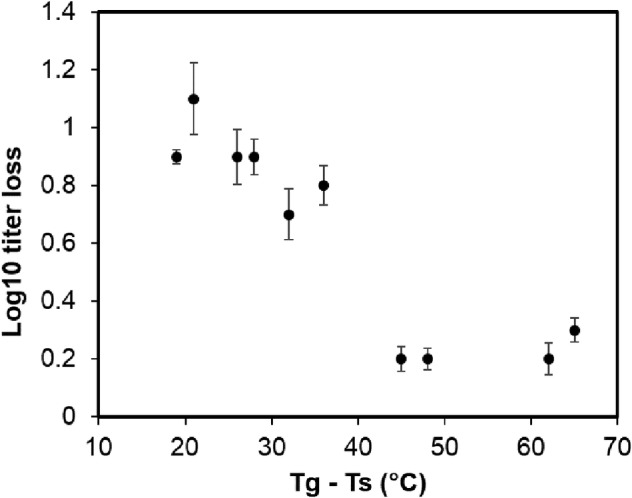
Scatterplot of difference between glass transition temperature (*T*_g_) and storage temperature (*T*_s_) and log_10_ titer loss of PEV20

Figure 7 shows the relationship between (*T*_g_ − *T*_s_) and phage stability. As the temperature gap between *T*_s_ and *T*_g_ increased, the phage titer loss was minimized. Phages seemed to remain biologically active in spray dried powders when stored at temperatures 40–50°C below the *T*_g_. No further improvement on phage stability was observed when (*T*_g_ − *T*_s_) exceeded 45°C, suggesting it is the threshold for storage stability of the phages in spray dried powders.

**FIGURE 8:**
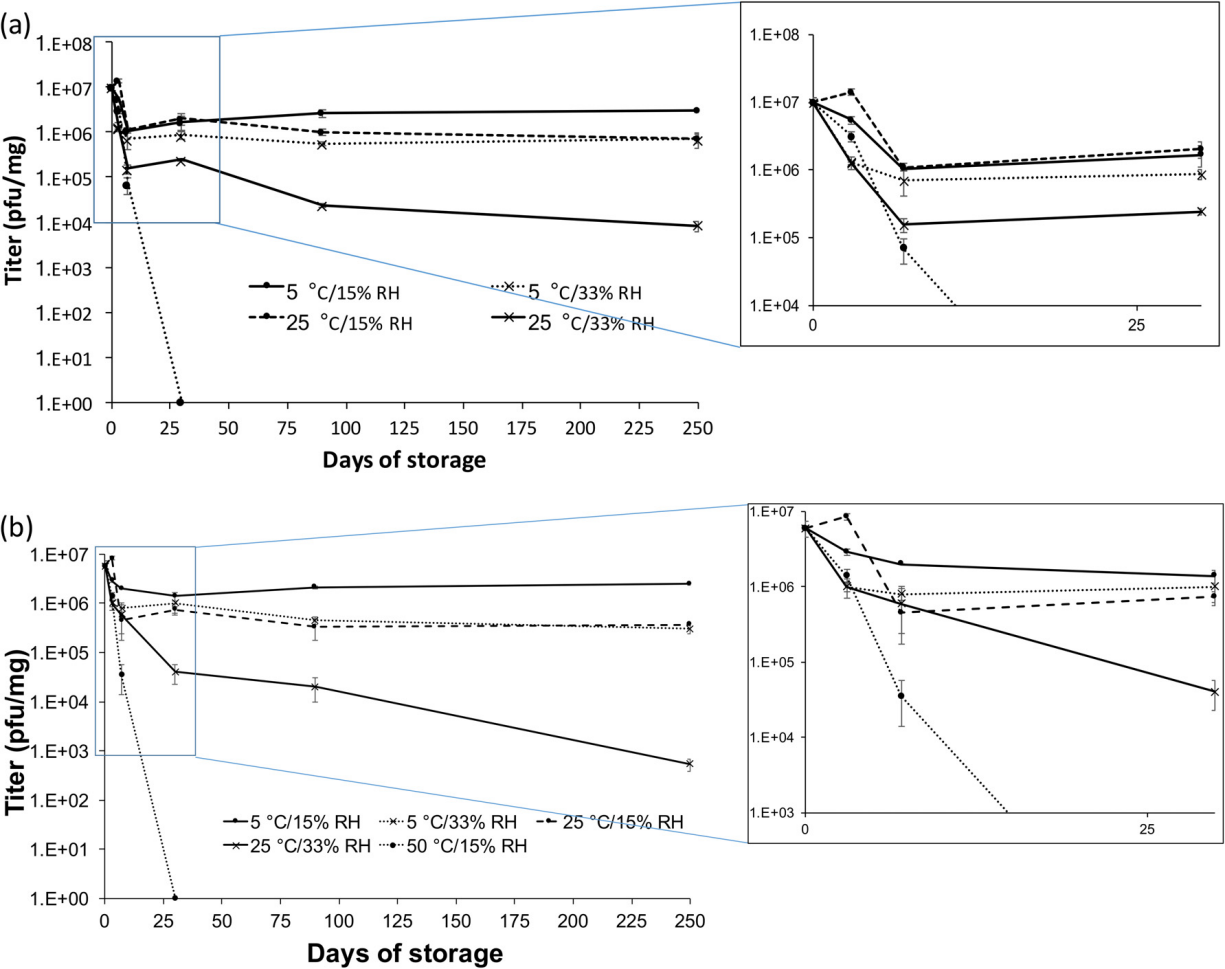
Titer of PEV20 powders containing 80% lactose/20% leucine (a) or 50% lactose/50% leucine (b) after storage at 5, 25, or 50°C and 15% RH or 33% RH (*n* = 3). Data are presented as mean ± SD in the graphs

To assess the effect of (*T*_g_ − *T*_s_) on long term stability of phages in powder formulations, phage powders were stored for 250 days. Phages remained stable (0.4–0.6 log_10_ titer loss) after 250 days of storage at 5°C/15% RH, where (*T*_g_ − *T*_s_) > 60°C (Figure 8). Storage at 5°C/33% RH and 25°C/15% RH, where (*T*_g_ − *T*_s_) is 26–48°C, caused ~1.1 log_10_ titer reduction at day 7 with no further loss. Significant titer reduction was observed (3–4 log_10_) after 250 days of storage at 25°C/33% RH, where (*T*_g_ − *T*_s_) is <20°C. Hence, storage stability of phages in spray dried powder is closely associated with (*T*_g_ − *T*_s_), it is important to either (a) increase the *T*_g_, or (b) store at a temperature sufficiently lower than *T*_g_. By slowing down the molecular mobility and rearrangement, phages can retain their original conformation and remain biologically active in the solid state. Furthermore, the residual water in spray dried powders may also assist in hydrating the phages in the solid state. In addition to *T*_s_, the use of smaller and molecularly flexible sugars was reported to better stabilize proteins.^33^ Sugars with flexible molecular structures can provide ease of molecular interaction with proteins, achieving a tighter packing with increased density.^10^ Increased density, in turn, decreases free volume and helps to immobilize proteins. These concepts for stabilizing proteins may also be applicable to phages. There are many different types of phages with different chemistry and structures. Currently, development of stable phage powders somewhat requires empirical efforts. The stability profile of spray dried phage powders may vary depending on the excipients used, which could lead to a lengthy development process. This study showed that (*T*_g_ − *T*_s_) is a key consideration which may help in the development of stable phage powder formulations.

## 4 CONCLUSIONS

This is the first study investigating the stabilization mechanism for phages in pharmaceutical solids. PEV20 phages were co‐spray dried with lactose and leucine to form partially crystalline particles. The *T*_g_ of the powders were manipulated by storing at different temperatures and relative humidity. It was found that phage viability was closely associated with the temperature gap between *T*_s_ and *T*_g_. The phages remained stable when the (*T*_g_ − *T*_s_) value was greater than 46°C, and phage inactivation was worsened with diminishing (*T*_g_ − *T*_s_) values. Similar effects were observed on powders containing 50 or 80% lactose. These findings corroborate with the vitrification hypothesis for phage stabilization and have significant implications in the production and storage of aerosol powders for inhaled phage therapy.

## 5 CONFLICT OF INTEREST

Authors do not have any conflict of interest to declare.

## Supporting information


**FIGURE S1**: X‐ray powder diffraction patterns of spray dried PEV20 powders containing 80% lactose and 20% leucine, and 50% lactose and 50% leucine after spray drying.
