# Supplementary figures and images for: Inhalable bacteriophage powders: Glass transition temperature and bioactivity stabilization
Source: Bioeng Transl Med. 2020 Apr 14;5(2):e10159. doi: 10.1002/btm2.10159 (PMC7237144; doi:10.1002/btm2.10159)

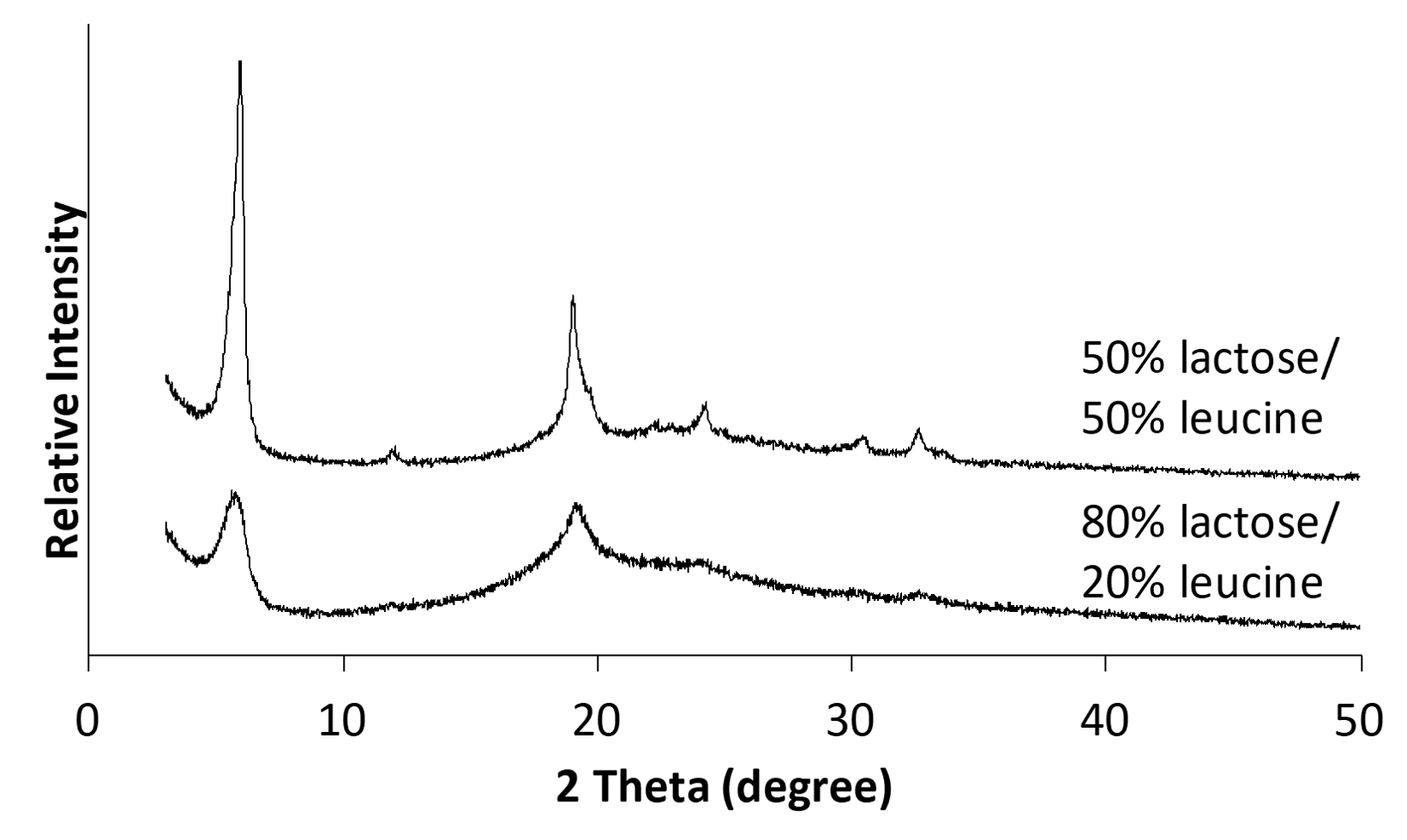

Supplement: Supplementary file 1 — FIGURE S1: X‐ray powder diffraction patterns of spray dried PEV20 powders containing 80% lactose and 20% leucine, and 50% lactose and 50% leucine after spray drying. [file BTM2-5-e10159-s001.tif]
